# Mitochondrial Genome Characteristics of *Rhodobryum laxelimbatum* and Comparative Analysis of RNA Editing and Codon Usage Bias With the Chloroplast Genome

**DOI:** 10.1002/ece3.74421

**Published:** 2026-10-14

**Authors:** Jingyuan Niu, Shuo Shi, Shuangling Li, Jiancheng Zhao, Lin Li

**Affiliations:** ^1^ School of Basic Medical Sciences Henan Medical University Xinxiang China; ^2^ College of Life Science Hebei Normal University Shijiazhuang China

**Keywords:** chloroplast genome, codon usage bias, comparative analysis, mitochondrial genome, *Rhodobryum laxelimbatum*, RNA editing

## Abstract

*Rhodobryum laxelimbatum* is a traditional medicinal plant belonging to the genus *Rhodobryum*. To date, studies on this genus have focused primarily on its geographical distribution, chemical constituents, and biological activities, whereas genomic data within the genus are limited. The mitochondrial genome of *R. laxelimbatum* was sequenced, assembled, and annotated. Genomic structural features and simple sequence repeats (SSRs) were comprehensively characterized. Comparative analyses of RNA editing patterns and codon usage bias were performed using the mitochondrial and chloroplast genomes of *R. laxelimbatum*. The results revealed that the complete mitochondrial genome of *R. laxelimbatum* was 103,071 bp in length. It comprises 40 protein‐coding genes (*Nad7* is a pseudogene), 24 transfer RNA genes, and 3 ribosomal RNA genes. In total, 76 SSRs were detected. The mitochondrial and chloroplast genomes contained 189 and 608 RNA editing sites, respectively, with the conversion of hydrophilic amino acids to hydrophobic amino acids being dominant. Both organellar genomes exhibited the highest GC content at the first codon position. GC12 was significantly associated with GC3 only in chloroplasts with steeper regression trends. Mitochondrial genes exhibited greater variation in the effective number of codons (ENC), and most genes in both genomes clustered near the expected ENC curve. Parity Rule 2 plot revealed a distinct base‐composition imbalance at synonymous third codon positions—mitochondria exhibited T3s > A3s with comparable G3s and C3s, whereas chloroplasts exhibited T3s > A3s and G3s > C3s. Five optimal codons were identified in the mitochondrial genome, predominantly ending in G/A/C, whereas nine were identified in the chloroplast genome, ending in A/U. Correspondence analysis demonstrated that codon usage bias was shaped by multiple interacting factors rather than a single determinant. This study provides crucial evidence for clarifying the evolutionary patterns of organelle genomes in *Rhodobryum*, and for understanding its genetic characteristics and molecular basis.

## Graphical abstract

This study notes that genomic‐level investigations of *Rhodobryum laxelimbatum* remain extremely limited. The mitochondrial genome assembled in this experiment, together with its comparative analysis against the chloroplast genome, offers key evidence to clarify organelle‐genome evolutionary patterns in *Rhodobryum* and to unravel the genetic traits and molecular underpinnings of this species.

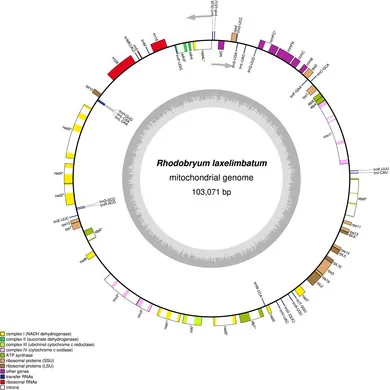

## 1 Introduction

*Rhodobryum laxelimbatum* belongs to the genus *Rhodobryum* (Bryaceae, Bryales, and Bryopsida). Most species of this genus are medicinal mosses. For example, *R. roseum* and *R. giganteum* are known as “Huixincao,” a well‐known Chinese medicinal herb (Harris and Bilun 2009; He et al. 2023). They contain various chemical constituents, such as amino acids, organic acids, phenolic compounds, and flavonoids (Yan et al. 2024; Sabharwal et al. 2023). It exhibits various pharmacological effects, including antioxidant, antibacterial, anti‐inflammatory, anti‐myocardial ischemia, lipid‐lowering, and anti‐atherosclerotic activities (Sabharwal et al. 2023; Pandith et al. 2025; Lei et al. 2001; Qiao et al. 2003). Additionally, they can be used for the clinical management of palpitations, coronary heart disease, hypertension, neurasthenia, and related disorders (He et al. 2023). Although direct experimental data on the pharmacological activities and pharmacodynamic effects of *R. laxelimbatum* are lacking, the existing literature indicates that it shares comparable traditional medicinal efficacy with its congeneric species ( *R. roseum* and *R. giganteum* ) and is also recognized as a crucial medicinal moss (Liu and Lin 2021; Yi 2000). To date, studies on plants of the genus have primarily focused on their geographical distribution, systematic taxonomy, chemical constituents, and pharmacological activities. However, limited attention has been given to these genomic studies.

The complete genomic data comprise three genetic components: nuclear, mitochondrial, and chloroplast genomes. The latter two are located within organelles and are collectively referred to as organelle genomes. Compared to nuclear genomes, organelle genomes are relatively simple in structure, making them the preferred focus of numerous studies. Additionally, they are essential for understanding, analyzing, and using genetic data. With rapid advances in molecular research, higher plants have become major experimental models, whereas genomic studies of mosses remain limited. As a model moss, *Physcomitrella patens* was the first moss species to have its organellar genomes fully analyzed (Terasawa et al. 2007; Sugiura 2003). Subsequently, the complete mitochondrial genomes of *Anomodon rugelii, Anomodon attenuatus, Atrichum angustatum, Bartramia pomiformis*, and other species were obtained, revealing high conservation in genome composition, gene order, and intron composition, while the available dataset of moss organelle genome data (Xue 2010; Liu et al. 2014).

Genome sequencing of species in the genus *Rhodobryum* can be traced back to 2019. Liu et al. (2019) used gene capture technology to sequence the protein‐coding regions of 142 plant nuclear genomes, including *Rhodobryum ontariense* , and elucidated the evolutionary dynamics of nuclear and plastid non‐synonymous substitutions and the phylogenetic relationships among major moss lineages. This study provides valuable molecular resources for the evolutionary studies of *Rhodobryum*. However, owing to the absence of fully assembled organellar genomes, a comprehensive and systematic assessment of organellar genomes evolution in *Rhodobryum* has not yet been achieved. Previously, we reported the complete chloroplast genome of *R. laxelimbatum* (Li et al. 2021). Subsequently, Shen et al. (2024) sequenced and analyzed the chloroplast genome of *Rhodobryum giganteum* . The phylogenetic results robustly placed *R. giganteum* within the order Bryales, forming a close sister relationship with *R. laxelimbatum*. However, mitochondrial genome data remain unavailable for any species in this genus.

The lack of available mitochondrial genome data and comparative analyses of dual organellar genomes has become a crucial bottleneck restricting genomic research on *Rhodobryum*. In this study, obtaining the mitochondrial genome of *R. laxelimbatum*, and comparing RNA editing and codon usage bias between its mitochondrial and chloroplast genomes will facilitate more systematic and in‐depth research on *Rhodobryum*. These results provide valuable insights for the subsequent clarification of the species' genetic background and further exploration of relevant regulatory mechanisms.

## 2 Materials and Methods

### 2.1 DNA Extraction, Sequencing, Mitochondrial Genome Data Assembly and Annotation

The specimen *R. laxelimbatum* was collected from Haba Snow Mountain, Shangri‐La, Yunnan, China (100.117°E, 27.313°N) and deposited in the Herbarium of Hebei Normal University (Shuo Shi, SZ5212; September 19, 2016). Whole moss individuals were collected and preserved using silica gel desiccation. Approximately 5 mg of green vegetative tissue was selected from the dried samples for DNA extraction, following the protocol described by Li et al. (2013) DNA fragments (300–500 bp) were selected to generate the sequencing libraries. Sequencing was performed on an Illumina HiSeq X Ten platform (Novogene Biotech Company, Beijing, China) using 150‐bp paired‐end reads, resulting in over 5.4 Gb of raw data. The raw sequencing data were subjected to quality assessment using FastQC v0.11.9. Adapter trimming and quality filtering were performed using fastp v0.20.1 with parameters ‐q 20 and ‐u 10, whereas all other parameters were set to default, resulting in clean data for subsequent analyses. The assembly was performed using the default settings of GetOrganelle v1.7.7.1 (Jin et al. 2020), with SPAdes and embplant_mt serving as the core assembler and seed reference database, respectively. No additional post‐assembly polishing steps were performed. The average coverage depth of the assembled mitochondrial genome was estimated to be 307.8×.

The complete mitochondrial genomes were annotated using Geneious Prime 2025.0.2 (https://www.geneious.com). *Mielichhoferia elongata* (GenBank accession no. NC_036945.1, mitochondrial genome), a closely related species, was selected as the reference sequence for annotating protein‐coding and rRNA genes, followed by thorough manual curation. The tRNA genes were predicted using tRNAscan‐SE v2.0 (Cox et al. 2004; Chan et al. 2021), and the predicted tRNA loci were further inspected in Geneious Prime. The final mitochondrial genome was visualized as a circular map using the OGDRAW software.

Basic genome characterization, including genome size, GC content, and gene coding regions, was performed using PhyloSuite v2 (Zhao et al. 2025). Putative protein‐coding genes within the intergenic spacer regions were identified using open reading frame (ORF) Finder (https://www.ncbi.nlm.nih.gov/orffinder/), and only ORFs ≥ 300 bp were retained for further analysis. To detect potential exogenous sequences within intergenic spacers, local BLASTN searches were conducted against a customized database of the model organism, using its chloroplast (NC_005087.1) and nuclear genomes (http://www.ncbi.nlm.nih.gov/genome/383) as references. The BLASTN parameters were set as follows: e‐value = 1e−5, max_target_seqs = 5, num_threads = 4, and outfmt = 6. Only aligned sequences ≥ 50 bp were subjected to statistical analysis.

### 2.2 SSR Analysis of Mitochondrial Genome Data

SSRs were identified using the MIcroSAtellite identification tool (https://pgrc.ipk‐gatersleben.de/misa). The minimum repeat thresholds were set as follows: mononucleotide, dinucleotide, trinucleotide, tetranucleotide, and hexanucleotide repeats ≥ 10, 12, 15, and 24 bp, respectively.

### 2.3 RNA Editing of Mitochondrial and Chloroplast Genomes

Potential RNA editing sites in the mitochondrial and chloroplast genomes of *R. laxelimbatum* (the chloroplast genome has been previously published; Li et al. 2021) were predicted using PREPACT v3.12.0 (Lenz et al. 2009) that relies on the NCBI BLAST 2.2.26+. The mitochondrial (NC_007945.1) and chloroplast (NC_005087.1) genomes of *P. patens* were used as references.

### 2.4 Codon Usage Bias Analysis

Coding sequences were extracted from the mitochondrial and chloroplast genome datasets using PhyloSuite v2. To minimize sampling bias, only genes possessing standard initiation codons and canonical termination codons were retained, and sequences < 300 bp were excluded from subsequent analyses (Jiang et al. 2008; Liu 2013). Stop codons (UAG, UAA, and UGA) were excluded from the analysis because each stop codon can only occur once in a single CDS sequence. The overall GC content of the CDS and the GC content at the 1st, 2nd, and 3rd codon positions (GC1, GC2, and GC3, respectively) were calculated using MEGA (Kumar et al. 2018). CodonW 1.4.2—with default parameters implemented as a plugin in PhyloSuite v2—was used for the statistical analysis of codon usage. For example, the base composition of synonymous third codon positions (A3s, T3s, G3s, and C3s), relative synonymous codon usage (RSCU), effective number of codons (ENC), codon adaptation index (CAI), frequency of optimal codons (Fop), length of amino acids (L‐aa), and grand average of hydropathy (Gravy) (Sharp and Li 1986; Wright 1990).

Primary methods included the following:

#### 2.4.1 Neutrality Plot Analysis

The neutrality plot was generated with GC3 and GC12 on the *x*‐ and *y*‐axes, respectively. An ordinary least‐squares simple linear regression was implemented in Microsoft Excel to produce the fitting trend‐line for this plot. Pearson's correlation analysis with a two‐tailed test was conducted to assess the correlation between GC12 and GC3 using the IBM SPSS Statistics 21 software.

#### 2.4.2 ENC–GC3s Plot Analysis

The expected value of ENC can be calculated using the following formula: ENC = 2 + GC_3_ + 29/(GC_3_^2^ + [1–GC_3_]^2^). A two‐dimensional scatter plot was generated with GC3s and ENC as the *x*‐ and *y*‐axes, respectively, demonstrating the standard curve for expected ENC values and the software‐calculated ENC values. To further quantify the discrepancy between the expected and observed ENC values, the ENC ratio was calculated as follows: (expected ENC – observed ENC)/expected ENC.

#### 2.4.3 Parity Rule 2 (PR2) Plot Analysis

This study analyzed the ATGC composition at the third position of amino acids encoded by four‐codon families, calculated the G3s/(G3s + C3s) and A3s/(A3s + T3s) ratios for each gene, and plotted these ratios as the *x*‐axis and *y*‐axis coordinates respectively. Subsequently, the distribution patterns of each gene were compared relative to the central point (*x* = 0.5, *y* = 0.5) in the coordinate plane.

#### 2.4.4 Selection of the Optimal Codon

To identify the optimal codons, all protein‐coding genes were sorted based on their ENC values. The top 10% of genes with the highest ENC and the bottom 10% with the lowest ENC values were used to construct the high‐ and low‐bias gene libraries, respectively. The RSCU values were computed separately in the two libraries. Codons with ΔRSCU ≥ 0.08 and RSCU > 1 in the high‐bias library were identified as optimal codons (Sharp and Li 1986; Wu et al. 2019; Zhang et al. 2024; Shen et al. 2025).

#### 2.4.5 Correspondence Analysis

To assess the variation trends of different gene codons, correspondence analysis (COA) based on the RSCU values of individual genes was performed using the CodonW program. Three termination codons, AUG (methionine) and UGG (tryptophan), were automatically excluded, and all genes were distributed in a 59‐dimensional vector space. The first and second major variation axes (Axis 1 and Axis 2) were used as the x‐ and y‐axes, respectively, to generate the COA scatter plot for visualizing the distribution and divergence of codon usage patterns among individual genes. Pearson's correlation analysis with a two‐tailed test was performed using the SPSS software to assess the associations between factor axes and codon‐bias–related parameters. The Benjamini–Hochberg false discovery rate procedure was used for *p*‐value adjustment to account for multiple comparisons.

## 3 Results

### 3.1 Mitochondrial Genome Features in *R. laxelimbatum*

The assembled mitogenome of *R. laxelimbatum* was 103,071 bp long. It contains 40 protein‐coding genes (among which *Nad7* is identified as a pseudogene), 24 transfer RNA (tRNA) genes, and three ribosomal RNA (rRNA) genes (Figure 1).

**FIGURE 1:**
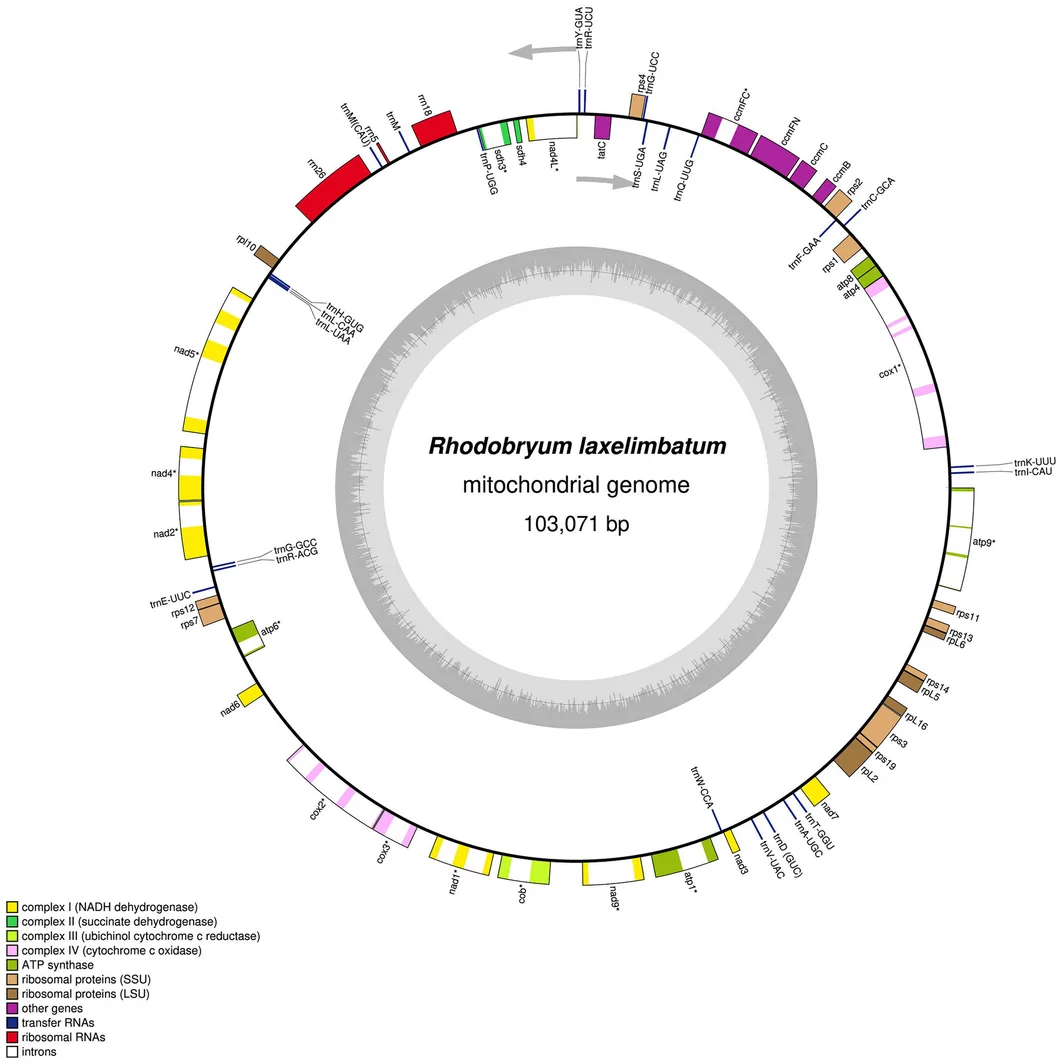
Circular map of mitochondrial genome from *R. laxelimbatum*. Genes inside and outside the circle are transcribed clockwise and counterclockwise, espectively. Genes from different functional groups are color‐coded. Inner circle: Dark gray represents GC content, and light gray represents AT content. (Circular map for schematic representation only, not the actual genome structure.)

The gene sequence was 70,419 bp, accounting for 68.32% of the total genome. The exons comprised 39,107 bp (37.94% of the genome), introns 31,312 bp (30.38%), and intergenic spacers 32,668 bp (31.69%). The GC content of the genes was 40.99%, slightly higher than that of the overall GC content (39.8%), whereas the intergenic regions exhibited a lower GC content (37.24%) (Table 1). Three pairs of overlapping genes were identified. Specifically, ATP synthase subunit 4 (*atp4*) and *atp8* overlapped by 8 bp, cytochrome c oxidase subunit I (*cox1*) and *atp4* overlapped by 4 bp, and ribosomal protein S12 and ribosomal protein S7 overlapped by 4 bp (Figure 2).

**TABLE 1:** The characteristics of *R. laxelimbatum* mitochondrial genome.

Genome size (bp)	103,071
Gene size (bp)	70,419
Proportion of genes (%)	68.32
Exon size (bp)	39,107
Proportion of exon (%)	37.94
Intron size (bp)	31,312
Proportion of intron (%)	30.38
Intergenic spacers size (bp)	32,668
Proportion of intergenic spacers (%)	31.69
GCtotal (%)	39.8
GCgenes (%)	40.99
GCgene spacers (%)	37.24

*Note:* When calculating the size of genes, pseudogenes and ORFs are not included, but the size of duplicate genes should be added. Gene spacers include pseudogenes and ORFs.

**FIGURE 2:**
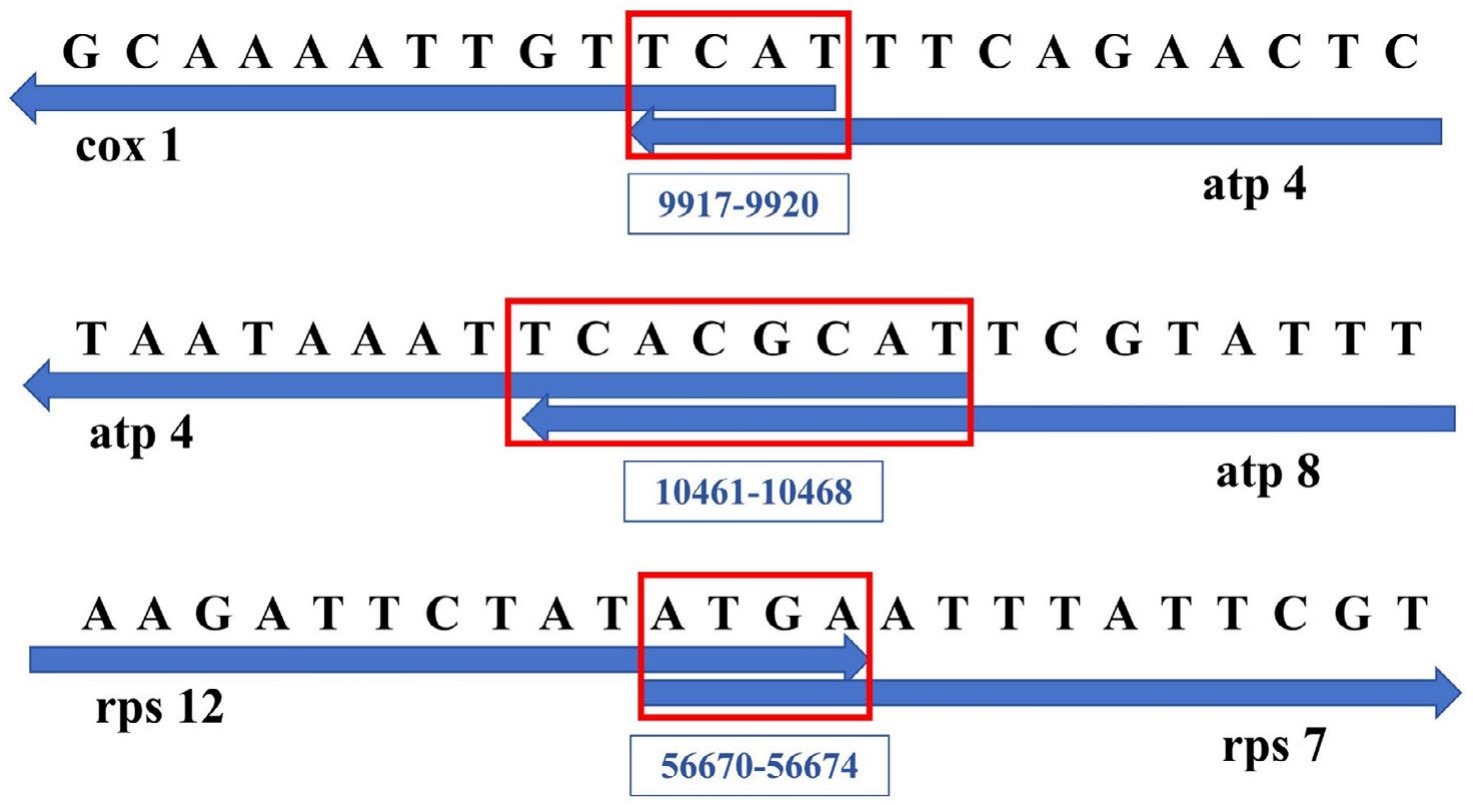
Three pairs of overlapping genes in the mitochondrial genome of *R. laxelimbatum*.

The mitochondrial genome harbors 3 Group I introns and 22 Group II introns (Dombrovska and Qiu 2004; Knoop 2004). Additionally, two ORFs ≥ 300 bp were present in the introns of *cox1* and NADH dehydrogenase subunit 5 (*nad5*) (Table 2).

**TABLE 2:** Introns content of mitochondrial genome.

Intron types	*atp1i1129g2 atp6i80g2 atp9i21g2 atp9i87g2 atp9i95g2* *ccmFCi829g2 cobi420g1 cox1i511g2 cox1i624g1 cox1i732g2* *cox1i1064g2 cox2i104g2 cox2i373g2 cox2i691g2 cox3i506g2* *nad1i287g2 nad1i728g2 nad2i156g2 nad4i461g2 nad4Li283g2* *nad5i230g2 nad5i753g1 nad5i1455g2 nad9i283g2 sdh3i100g2*	
ORF	ORF622	1869 bp (in *cox1*)
	ORF533	1602 bp (in *nad5*)

*Note:* Intron nomenclature follows Dombrovska and Qiu (2004) and Knoop (2004).

The mitochondrial genome contained 64 intergenic regions, of which 38 were ≥ 300 bp. Notably, no ORFs were detected within these regions, a result significantly different from the observations reported for intergenic spacers in other mosses (Liu et al. 2014).

A total of 48 intergenic spacers ≥ 50 bp were analyzed. Local BLASTN searches revealed no foreign sequences of chloroplast origin satisfying the above criteria. In contrast, 26 nuclear‐derived sequences were identified, encompassing 8625 bp and representing 8.37% of the complete mitochondrial genome (Table 3).

**TABLE 3:** The transfer sequences of mitochondrial intergenic spacers.

Chloroplast sequence	Number	0
	Length	0 bp
	Proportion	0%
Nuclear sequence	Number	26
	Length	8625 bp
	Proportion	8.37%

In total, 76 SSRs were identified. Mononucleotide repeats were the most abundant (34, 44.74%), followed by dinucleotide (23, 30.26%), tetranucleotide (11, 14.47%), complex (5, 6.58%), trinucleotide (2, 2.63%), and pentanucleotide repeats (1, 1.32%). The mononucleotide repeats were predominantly composed of A and T bases, whereas dinucleotide repeats were primarily composed of (AT) motifs (Figure 3). (AT)_*n*_ was the dominant SSR type, a common feature observed in numerous plant species (Varshney et al. 2005; Powell et al. 1996).

**FIGURE 3:**
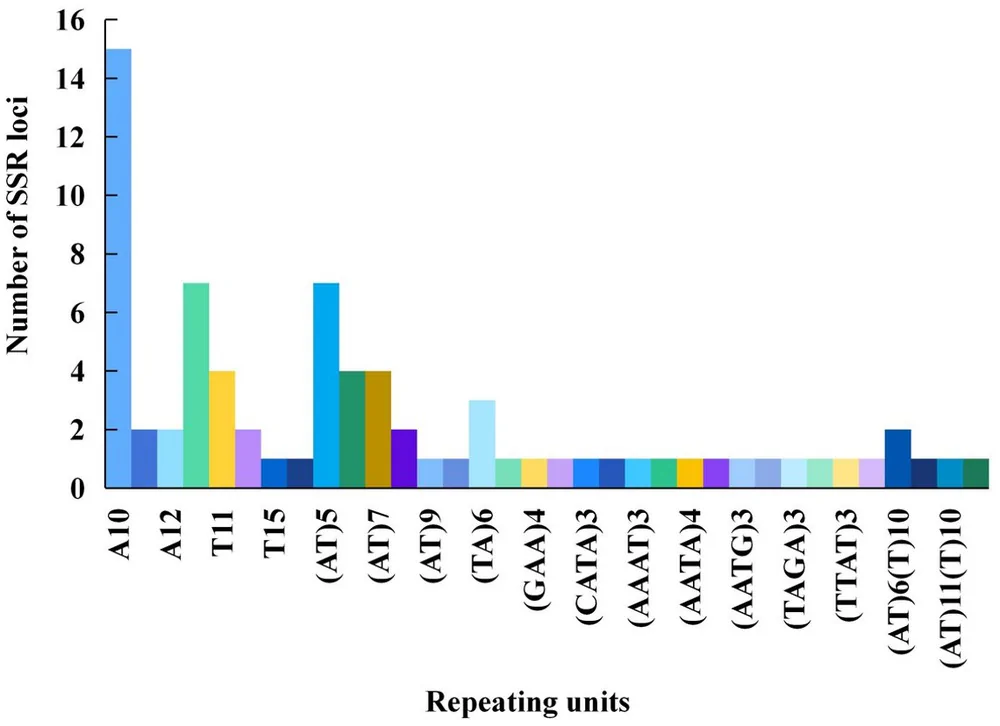
Distribution of SSRs in the mitochondrial genome.

### 3.2 RNA Editing Sites in Chloroplast and Mitochondrial Genomes

The mitochondrial genome contained a total of 189 RNA editing sites, with the majority attributed to C‐to‐U transitions (128 sites). The distribution of these editing sites varied significantly among the different genes. Notably, the cytochrome c biogenesis‐related mitochondrial genes *ccmFc* and *ccmFN* exhibited relatively high numbers of editing sites, followed by *nad5* and *sdh3*. In the chloroplast genome, a significantly higher number of RNA editing sites was predicted (608 sites). Among these, the numbers of C‐to‐U and U‐to‐C editing events were comparable, with 318 and 290 sites, respectively. The genes hypothetical chloroplast reading frame 2 (*ycf2*) and *ycf1* exhibited the highest frequencies of editing sites, followed by RNA polymerase beta subunit 2 and maturase K. In both the mitochondrial and chloroplast genomes, 28 types of amino acid transitions were identified, with the conversion from hydrophilic to hydrophobic amino acids being predominant (Figure 4).

**FIGURE 4:**
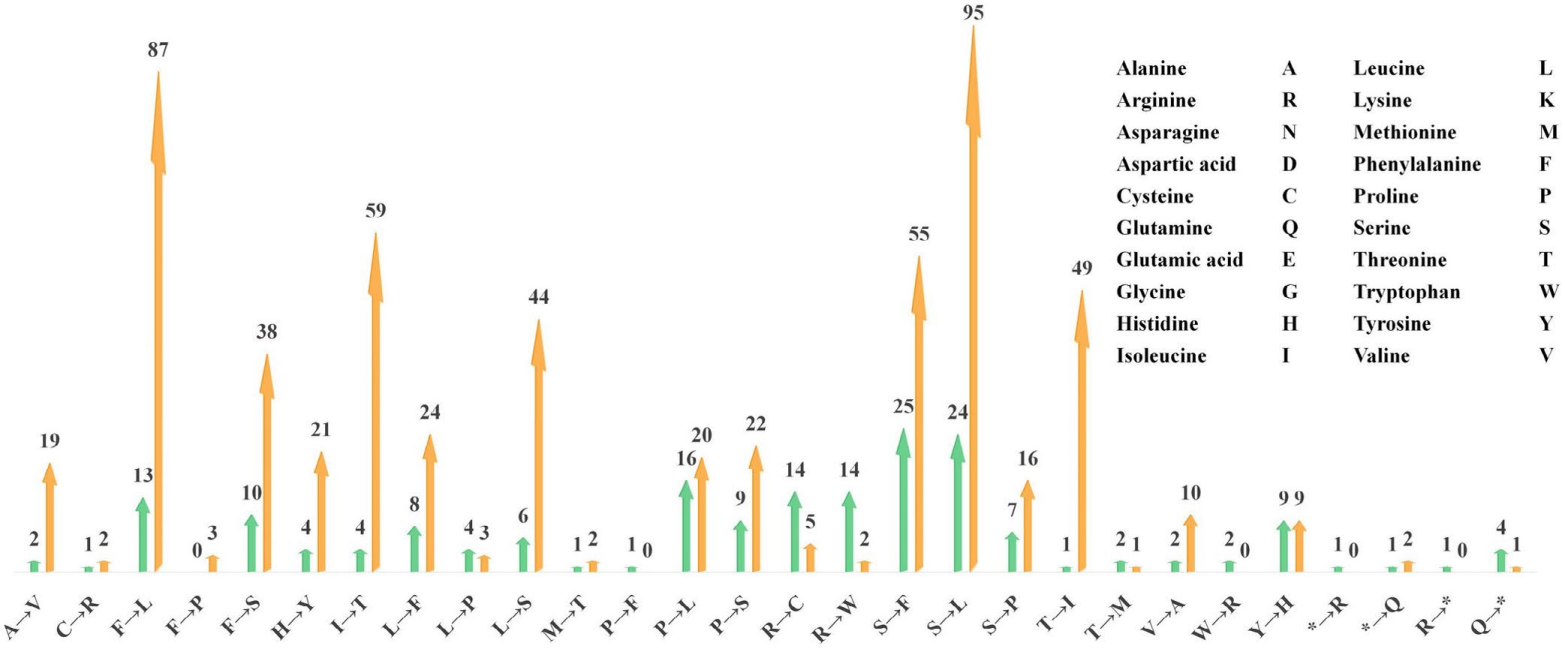
Amino acid changes after organelle genomes RNA editing. * stands for termination codon. Green and orange arrows indicate the amino acid substitution types of the mitochondrial and chloroplast genomes, respectively.

### 3.3 Analysis of Codon Bias and Influencing Factors in Chloroplast and Mitochondrial Genomes

Twenty‐eight mitochondrial and 52 chloroplast protein‐coding sequences were subjected to codon usage bias analysis. Both the mitochondrial and chloroplast genomes exhibited the highest GC content at the first codon position, followed by the second position, whereas the third codon position possessed the lowest GC content. Overall, the codon usage of protein‐coding genes in the organellar genomes of R. laxelimbatum demonstrated a preference for A/T (Table 4).

**TABLE 4:** GC composition of *R. laxelimbatum* organelle genome.

	GC1 (%)	GC2 (%)	GC3 (%)	GCall (%)
Mitochondrial genome	41.3	37.63	28.67	35.86
Chloroplast genome	40.77	34.47	14.91	30.05

#### 3.3.1 Analysis of Neutrality Plot

In the mitochondrial genome, the GC_3_ content of individual genes exhibited a relatively narrow distribution, ranging from approximately 0.175–0.318 (Figure 5). The genes were not distributed along or near the diagonal line. The regression curve between GC_3_ and GC_12_ was nearly parallel. The correlation coefficient between GC_12_ and GC_3_ was 0.018. Two‐tailed testing revealed no significant correlation, indicating differences in base composition among the first, second, and third codon positions of the mitochondrial protein‐coding genes (Table 5).

**FIGURE 5:**
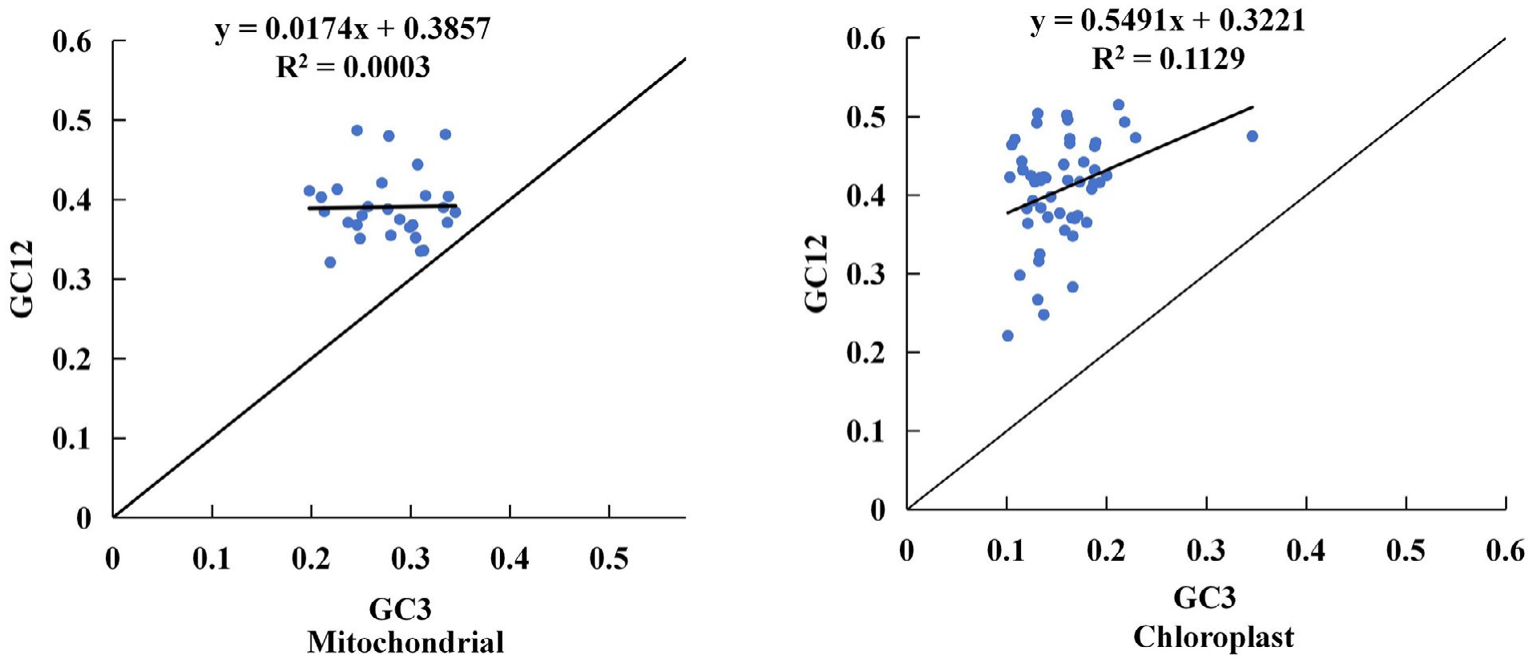
The neutrality plots between GC12 and GC3.

**TABLE 5:** Correlation coefficients and double‐tailed test results between GC12 and GC3.

	Pearson correlation (*r*)	Two‐tailed *p*	*t*	df
Mitochondrial genome	0.018	0.928	0.092	26
Chloroplast genome	0.336*	0.015	2.523	50

* Indicates significant correlation at *p* < 0.05 (two‐tailed).

In the chloroplast genome, the GC_3_ content varied from 0.074–0.302 (Figure 5) that was considerably lower than that in the mitochondrial genome. Among all the analyzed genes, the photosystem II reaction center protein D1 gene (psbA) exhibited a significantly higher GC_3_ value (0.346) than that of the other genes. The correlation coefficient between GC_12_ and GC_3_ was 0.336, with a two‐tailed *p‐*value < 0.05, indicating a significant correlation between GC_12_ and GC_3_ (Table 5).

#### 3.3.2 Analysis of ENC‐Plot

For both the mitochondrial and chloroplast genomes, most ENC values were distributed on or near the expected ENC curve derived from the GC_3_ composition; however, several genes fell below the expected curve (Figure 6). The distribution patterns of the ENC ratio were similar between the two organelles: the largest proportion of values ranged from −0.05 to 0.05, followed by those distributed between 0.05 and 0.15 (Table 6). Overall, these differences were relatively small. Notably, the distribution range of ENC values was wider in the mitochondrial genome, with nearly all ENC values above 35. In contrast, chloroplast ENC values exhibited a narrower distribution range and smaller variation in codon bias but contained significantly more genes with ENC values below 35 than the mitochondrion (Figure 6).

**FIGURE 6:**
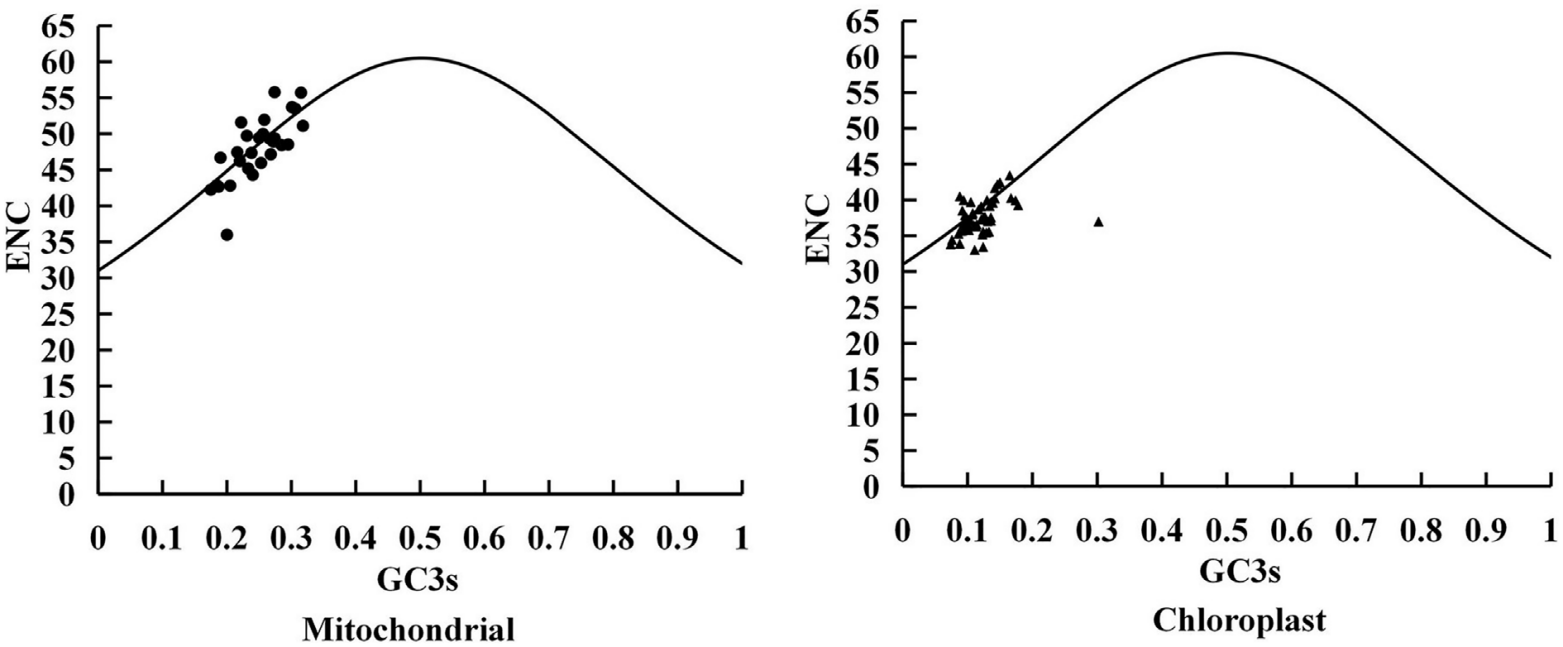
Analysis of ENC and GC3 relationship.

**TABLE 6:** Distribution of the ENC ration.

	Class limits	−0.15~−0.05	−0.05~0.05	0.05~0.15	0.15~0.3	Total
	Class midvalue	−0.1	0	0.1	0.23	
Mitochondrial	Frequency number	5	16	6	1	28
	Frequency	0.179	0.571	0.214	0.036	1
Chloroplast	Frequency number	3	34	14	1	52
	Frequency	0.058	0.654	0.269	0.019	1

#### 3.3.3 Analysis of Codon Usage Using PR2‐Plot

To discuss the divergence of AT and GC contents under balanced conditions, amino acids with exactly four synonymous codons were screened in this study, and the base usage deviation at the third codon position was analyzed. Under the sole effect of mutational pressure, the four nucleotides (A, T, C, and G) at the third codon position should have equal usage probability, with the theoretical values of *A3s/(A3s + T3s)* and *G3s/(G3s + C3s)* both being 0.5.

In the mitochondrial genome, most points were in the lower half of the area along the vertical direction, revealing that the usage frequency of T was higher than that of A at the synonymous position. However, horizontally, gene counts exhibited only minor differences between the left and right regions, revealing that the frequencies of G and C were similar at the synonymous positions (Figure 7).

**FIGURE 7:**
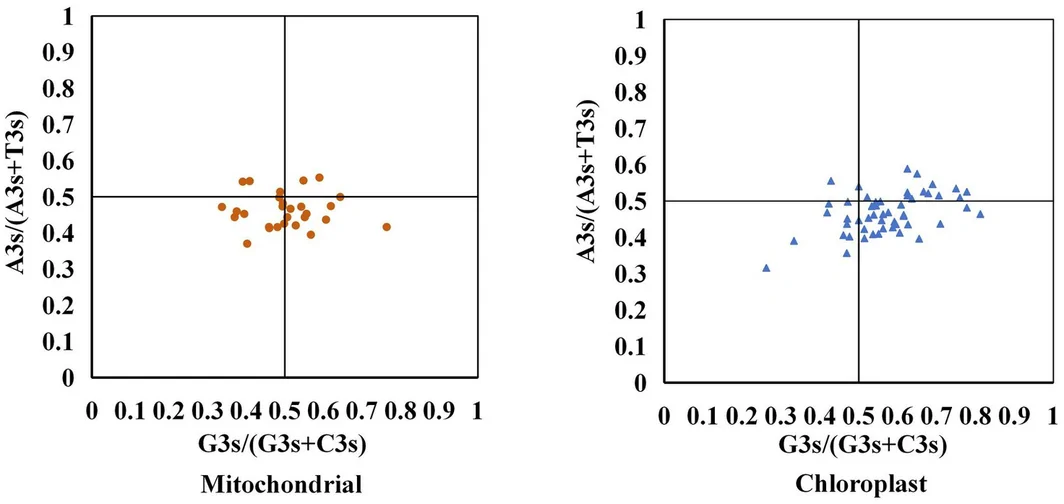
PR2‐bias Plot Analysis.

Similar distribution characteristics along the vertical direction were observed for chloroplast genes. However, horizontally, the right region exhibited significantly more gene scatter points than that of the left region, reflecting a significant bias toward T > A and G > C at the third codon position (Figure 7).

#### 3.3.4 Selection of Optimal Codons

Five optimal codons were obtained for the mitochondrial genes (UUG, CCC, AAA, AGC, and GGC). In contrast, nine optimal codons were identified in the chloroplast genome (Table 7). All chloroplast optimal codons ended in A or U, consistent with the general pattern observed in land plant chloroplasts.

**TABLE 7:** The optimal codons screening of mitochondrial and chloroplast genomes.

		Mitochondrial genome					Chloroplast genome				
		High expressed gene		Low expressed gene		ΔRSCU	High expressed gene		Low expressed gene		ΔRSCU
AA	Codon	Frequency	RSCU	Frequency	RSCU		Frequency	RSCU	Frequency	RSCU	
Phe	UUU	78	1.59	40	1.6	−0.01	32	1.78	220	1.88	−0.1
	UUC	20	0.41	10	0.4	0.01	4	0.22	14	0.12	0.1
Leu	UUA	50	2.08	43	2.84	−0.76	27	2.89	302	4.42	−1.53
	UUG	34	1.42	18	1.19	0.23*	5	0.54	20	0.29	0.25
	CUU	18	0.75	15	0.99	−0.24	17	1.82	61	0.89	0.93*
	CUC	11	0.46	1	0.07	0.39	3	0.32	6	0.09	0.23
	CUA	15	0.63	10	0.66	−0.03	4	0.43	19	0.28	0.15
	CUG	16	0.67	4	0.26	0.41	0	0	2	0.03	−0.03
Ile	AUU	51	1.61	42	1.7	−0.09	36	1.4	201	1.84	−0.44
	AUC	14	0.44	12	0.49	−0.05	8	0.31	9	0.08	0.23
	AUA	30	0.95	20	0.81	0.14	33	1.29	117	1.07	0.22*
Met	AUG	23	1	22	1	0	19	1	51	1	0
Val	GUU	26	1.7	25	1.64	0.06	11	1.69	55	1.65	0.04
	GUC	11	0.72	4	0.26	0.46	1	0.15	5	0.15	0
	GUA	17	1.11	25	1.64	−0.53	13	2	67	2.02	−0.02
	GUG	7	0.46	7	0.46	0	1	0.15	6	0.18	−0.03
Ser	UCU	34	1.58	14	2.1	−0.52	18	2.12	88	2.21	−0.09
	UCC	15	0.7	2	0.3	0.4	5	0.59	14	0.35	0.24
	UCA	28	1.3	4	0.6	0.7	8	0.94	77	1.93	−0.99
	UCG	8	0.37	4	0.6	−0.23	7	0.82	7	0.18	0.64
Pro	CCU	17	1.36	11	1.83	−0.47	11	1.69	54	2.43	−0.74
	CCC	14	1.12	4	0.67	0.45*	6	0.92	7	0.31	0.61
	CCA	12	0.96	7	1.17	−0.21	8	1.23	26	1.17	0.06
	CCG	7	0.56	2	0.33	0.23	1	0.15	2	0.09	0.06
Thr	ACU	18	1.26	15	2.4	−1.14	15	2.07	73	2.13	−0.06
	ACC	13	0.91	2	0.32	0.59	2	0.28	12	0.35	−0.07
	ACA	17	1.19	7	1.12	0.07	10	1.38	43	1.26	0.12*
	ACG	9	0.63	1	0.16	0.47	2	0.28	9	0.26	0.02
Ala	GCU	31	1.61	25	2.33	−0.72	19	1.55	62	2.07	−0.52
	GCC	9	0.47	3	0.28	0.19	6	0.49	8	0.27	0.22
	GCA	21	1.09	11	1.02	0.07	20	1.63	49	1.63	0
	GCG	16	0.83	4	0.37	0.46	4	0.33	1	0.03	0.3
Tyr	UAU	43	1.56	23	1.84	−0.28	25	1.72	145	1.86	−0.14
	UAC	12	0.44	2	0.16	0.28	4	0.28	11	0.14	0.14
TER	UAA	0	0	0	0	0	0	0	0	0	0
	UAG	0	0	0	0	0	0	0	0	0	0
His	CAU	18	1.38	4	1.6	−0.22	11	2	30	1.82	0.18*
	CAC	8	0.62	1	0.4	0.22	0	0	3	0.18	−0.18
Gln	CAA	44	1.6	16	1.68	−0.08	20	1.9	117	1.84	0.06
	CAG	11	0.4	3	0.32	0.08	1	0.1	10	0.16	−0.06
Asn	AAU	57	1.5	25	1.85	−0.35	33	1.89	264	1.75	0.14*
	AAC	19	0.5	2	0.15	0.35	2	0.11	38	0.25	−0.14
Lys	AAA	81	1.67	20	1.54	0.13*	45	1.8	366	1.93	−0.13
	AAG	16	0.33	6	0.46	−0.13	5	0.2	14	0.07	0.13
Asp	GAU	33	1.69	14	1.65	0.04	16	1.88	93	1.79	0.09*
	GAC	6	0.31	3	0.35	−0.04	1	0.12	11	0.21	−0.09
Glu	GAA	33	1.29	16	1.68	−0.39	27	1.86	156	1.85	0.01
	GAG	18	0.71	3	0.32	0.39	2	0.14	13	0.15	−0.01
Cys	UGU	16	1.28	6	2	−0.72	6	1.71	11	1.83	−0.12
	UGC	9	0.72	0	0	0.72	1	0.29	1	0.17	0.12
TER	UGA	0	0	0	0	0	0	0	0	0	0
Trp	UGG	29	1	8	1	0	12	1	49	1	0
Arg	CGU	20	1.4	8	1.66	−0.26	15	1.8	41	1.88	−0.08
	CGC	12	0.84	3	0.62	0.22	4	0.48	9	0.41	0.07
	CGA	13	0.91	8	1.66	−0.75	14	1.68	29	1.33	0.35*
	CGG	12	0.84	2	0.41	0.43	3	0.36	4	0.18	0.18
Ser	AGU	21	0.98	15	2.25	−1.27	13	1.53	45	1.13	0.4*
	AGC	23	1.07	1	0.15	0.92*	0	0	8	0.2	−0.2
Arg	AGA	16	1.12	6	1.24	−0.12	12	1.44	43	1.97	−0.53
	AGG	13	0.91	2	0.41	0.5	2	0.24	5	0.23	0.01
Gly	GGU	17	1.17	20	2	−0.83	15	1.58	53	1.84	−0.26
	GGC	15	1.03	7	0.7	0.33*	2	0.21	11	0.38	−0.17
	GGA	16	1.1	12	1.2	−0.1	16	1.68	46	1.6	0.08*
	GGG	10	0.69	1	0.1	0.59	5	0.53	5	0.17	0.36

*Note:* Codon entries with asterisked ΔRSCU values are defined as optimal codons, satisfying two criteria: ΔRSCU ≥ 0.08 and RSCU > 1 in the corresponding high‐expression gene set of each organelle (mitochondrion or chloroplast).

#### 3.3.5 Correspondence Analysis

Correspondence analysis was performed based on the relative RSCU values of the mitochondrial and chloroplast protein‐coding genes. For the mitochondrial genome, the first four principal axes accounted for 12.57, 10.94, 9.01, and 8.83 of the total variation, respectively, with a cumulative contribution of 41.35%. For the chloroplast genome, the first four axes also represented the major sources of variation, with contributions of 15.06, 7.54, 7.32, and 5.87, respectively, yielding a cumulative proportion of 35.79%. The contributions of subsequent axes gradually declined.

To further explore the influencing factors, the genes in both organelles were classified based on their functional categories. In the mitochondrial genome, a scatter plot of the first two principal axes revealed that most ribosomal protein‐coding genes were distinctly separated from their origin (Figure 8).

**FIGURE 8:**
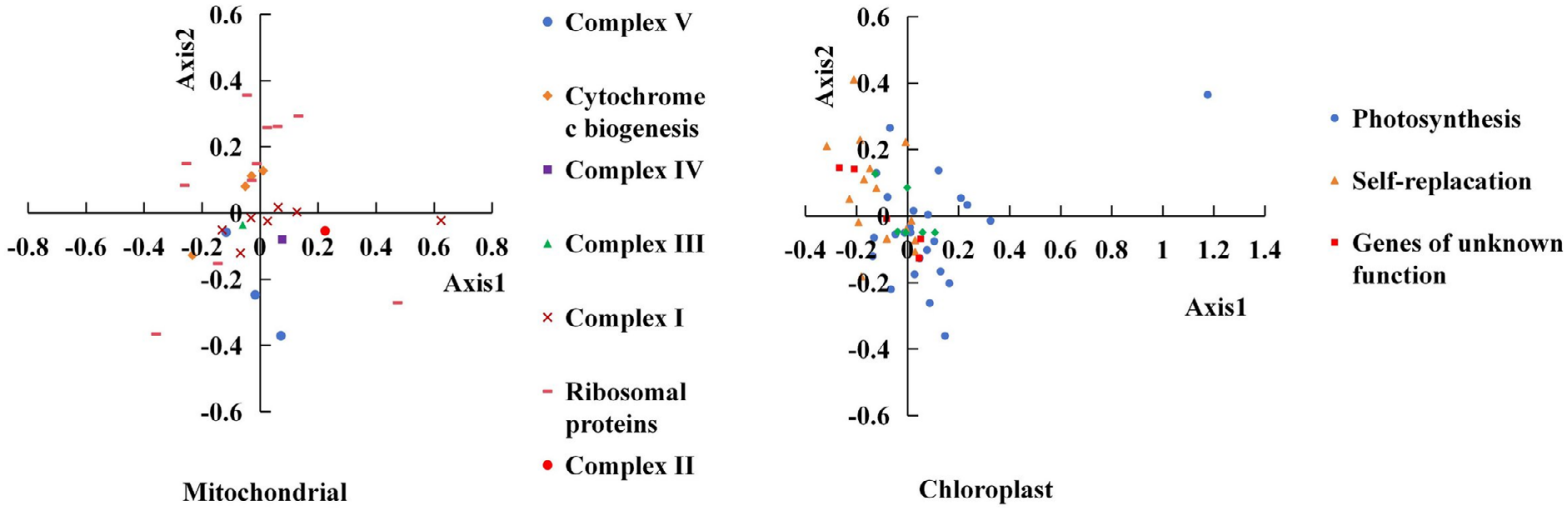
Corresponding analysis results based on relative synonymous codon usage.

In the chloroplast genome, the distribution pattern along axes 1 and 2 was similar, with most genes concentrated near the zero coordinate. Notably, *psbA* deviated significantly from the majority of genes, indicating that its codon usage bias differed from that of other genes (Figure 8).

Correlation analyses were performed between the first two principal axes and indices, including CAI, Fop, ENC, GC3s, GC, L‐aa, Gravy, and Aromo (Table 8). In the mitochondrial genome, the second axis was significantly correlated with the ENC and GC3s.

**TABLE 8:** Correlation among main parameters of gene codons.

		CAI	Fop	ENC	GC3s	GC	L_aa	Gravy	Aromo
**Mitochondrial**									
Axis1	*r*	0.235	0.071	0.153	0.237	0.185	−0.122	0.253	0.063
	Test statistic	1.233	0.363	0.789	1.244	0.96	−0.627	1.333	0.322
	df	26	26	26	26	26	26	26	26
	Raw *p*	0.229	0.718	0.437	0.225	0.345	0.537	0.195	0.752
	FDR‐adjusted *p*	0.523	0.752	0.636	0.523	0.552	0.677	0.523	0.752
Axis2	*r*	0.091	0.24	**0.622** *	**0.66** *	0.199	0.205	−0.357	0.118
	Test statistic	0.466	1.261	4.05	4.48	1.035	1.068	−1.949	0.606
	df	26	26	26	26	26	26	26	26
	Raw *p*	0.646	0.219	< 0.001	< 0.001	0.311	0.296	0.062	0.55
	FDR‐adjusted *p*	0.738	0.523	< 0.001	< 0.001	0.552	0.552	0.331	0.677
**Chloroplast**									
Axis1	*r*	**0.757** *	**0.679** *	0.006	**0.754** *	**0.399** *	0.098	0.285	0.276
	Test statistic	8.192	6.54	0.042	8.117	3.077	0.696	2.102	2.03
	df	50	50	50	50	50	50	50	50
	Raw *p*	< 0.001	< 0.001	0.968	< 0.001	0.003	0.491	0.04	0.047
	FDR‐adjusted *p*	< 0.001	< 0.001	0.968	< 0.001	0.01	0.604	0.091	0.094
Axis2	*r*	0.019	0.188	0.085	0.324	0.194	−0.103	**−0.405** *	−0.222
	Test statistic	0.134	1.353	0.603	2.422	1.398	−0.732	−3.132	−1.61
	df	50	50	50	50	50	50	50	50
	Raw *p*	0.893	0.181	0.551	0.019	0.168	0.468	0.003	0.114
	FDR‐adjusted *p*	0.953	0.263	0.63	0.051	0.263	0.604	0.01	0.203

*Note:* Bold values represent Pearson correlation coefficients.

* Significant at FDR‐adjusted *p* < 0.05 (Benjamini‐Hochberg correction).

In the chloroplast genome, the first axis was significantly correlated with CAI, Fop, GC3s, and GC, whereas axis 2 exhibited a significant negative correlation with the Gravy value.

## 4 Discussion

### 4.1 Characteristics of Mitochondrial Genome in *R. laxelimbatum*

The published *R. laxelimbatum* mitochondrial genome was 103,071 bp length, with a GC content of 39.8%. This was similar to that observed in previously sequenced plant mitochondrial genomes, such as those of *P. patens* (105,340 bp, GC 40.6%) (Terasawa et al. 2007) and *A. rugelii* (104,239 bp, GC 41.2%) (Xue 2010). Additionally, the number of genes was consistent with that of *P. patens* (67 genes), *A. rugelii* (67 genes), and other mosses reported in previous studies (Terasawa et al. 2007; Xue 2010; Liu et al. 2014).

The *nad7* gene encodes the *nad7* subunit of mitochondrial respiratory chain complex I (Bonen et al. 1994). Bell et al. (2014) discovered *nad7* pseudogeneization in the mitochondrial genome of *Tetraphis pellucida* and proposed that such pseudogenization events evolved independently in liverworts, mosses, and hornworts. Additionally, Liu et al. (2014) also detected *nad7* pseudogeneization in basal moss lineages; however, *nad7* pseudogeneization or gene loss was absent in non‐basal species.

*R. laxelimbatum* is a non‐basal moss species belonging to the Bryales family. Accordingly, *nad7* pseudogenization was not expected to occur in this species; however, our results confirmed the presence of *nad7* pseudogenization in *R. laxelimbatum*. This finding is consistent with that of a previous report by Goruynov et al. (2018), who documented *nad7* pseudogenization in another non‐basal moss species, *M. elongata*.

Because *M. elongata* , *T. pellucida* , and *Buxbaumia aphylla* are phylogenetically distantly related, and the exon structure of *nad7* in *M. elongata* differs significantly from that in *T. pellucida* and *B. aphylla* , together with the occurrence of *nad7* pseudogenization and gene loss in liverworts and hornworts (Xue 2010; Groth‐Malonek et al. 2007; Li et al. 2009), Goryunov hypothesized that the functional loss of the *nad7* gene evolved at least twice during moss evolution. The identification of *nad7* pseudogenization in the *R. laxelimbatum* mitochondrial genome in this present study further supports the evolutionary hypothesis proposed by Goryunov.

In previous studies, 27 introns were conserved and shared by 12 moss species, except for *Hypnum imponens* , that was deficient in nad7i209g2 (Liu et al. 2014). The intron composition identified in this study was largely consistent with that reported for other mosses. Only two introns (nad7i140g2 and nad7i209g2) were absent in *R. laxelimbatum*, a result closely associated with the functional impairment of the *nad7* gene. Notably, the introns (cox1i323g2 and cox1i1200g2) present in *Sphagnum* species and rrn18i839g1 detected in *Sphagnum palustre* , *Atrichum angustatus*, *T. pellucida* , and *Buxbaumia aphylla* , were absent from other moss lineages (Liu et al. 2014), including *R. laxelimbatum* examined in this study. According to existing systematic classifications, *S. palustre* , *A. angustatus* , *T. pellucida* , and *B. aphylla* belong to the basal–moss lineage. Additionally, these introns are present in the mitochondrial genome of liverworts. Collectively, it is reasonable to infer that these intronic elements were gradually lost during the evolutionary diversification of mosses.

No chloroplast‐derived exogenous sequences were identified in the mitochondrial intergenic regions of *R. laxelimbatum*, consistent with the results of previous studies (Terasawa et al. 2007; Liu et al. 2014; Bhattacharya et al. 2011). Nuclear‐derived sequences accounted for 8.37% and this portion was significantly lower than those reported for angiosperms and other moss species (Liu et al. 2014; Goremykin et al. 2012; Rodríguez‐Moreno et al. 2011). The accumulation of nuclear inserted fragments is strongly correlated with the genetic affinity between the target species and reference genomes. Additionally, the proportion would increase significantly when nuclear genomic data from Bryales species were used as the reference.

Repetitive sequences are the primary drivers of mitogenome rearrangement, with repeats ≥ 100 bp demonstrating higher recombination activity (Xu et al. 2026). Intronic repeats usually cause deleterious gene mutations, whereas adaptive rearrangements primarily occur in intergenic regions (Khan et al. 2019; Vieira et al. 2014). In *R. laxelimbatum*, mitochondrial repeats are intron‐limited and ≤ 100 bp, indicating weak rearrangement potential.

Longer repeats confer more SSR polymorphism (Temnykh et al. 2001; Yi et al. 2006; Liu et al. 2026). Despite frequent long motifs, mononucleotide repeats are unsuitable for SSR screening because of the frequent PCR slippage and genotyping errors (Kelkar et al. 2008). Higher organisms primarily contain mononucleotide SSRs, followed by di‐ and trinucleotide repeats (Nicot et al. 2004; Xue et al. 2019; Li et al. 2026), consistent with the SSR distribution in *R. laxelimbatum*. Based on previous results, researchers have summarized the SSRs that are more suitable for primer design: eight or more dinucleotide repeats, six or more trinucleotide repeats, five or more tetranucleotide repeats, and four or more pentanucleotide repeats (Cheng and Huang 2011). Complex long‐repeat SSRs were selected as the candidates. Based on these criteria, seven reliable SSR loci were identified in *R. laxelimbatum*. This feasible screening method greatly facilitates SSR mining in mosses under limited conditions.

### 4.2 The Occurrence of RNA Editing During the Evolution of Organellar Genomes

The predicted number of RNA editing sites in the chloroplast genome was significantly higher than that in the mitochondrial genome. This result contradicts the conventional understanding that “mitochondrial editing sites are generally more numerous than chloroplast ones” in terrestrial plants (Zhang et al. 2019; Gray 2009; Oldenkott et al. 2014; Zhang et al. 2025) and also exhibits significant differences from the editing characteristics of most moss species. For the model bryophyte *P. patens* , only 11 mitochondrial editing sites and 1 chloroplast editing site have been annotated in previous studies (Rüdinger et al. 2009; Takenaka et al. 2013). Dong et al. systematically explored the organellar RNA editing characteristics across bryophytes and confirmed distinct taxon‐specific editing patterns. Notably, no RNA editing sites were identified in Marchantiopsida; most other bryophyte lineages exhibited more editing events in the mitochondria than in chloroplasts. In these reported species, the number of mitochondrial editing sites was generally ≤ 400, whereas the number of chloroplast editing sites was ≤ 300 (Dong et al. 2019). The chloroplast genome of *Tortula atrovirens* contains only six RNA editing sites. In contrast, the mitogenome of *T. atrovirens* contains 51 RNA editing sites (Ma et al. 2024).

Although the PERFACT tool relies on homologous reference sequences and has inherent predictive limitations, the quantitative discrepancy is unlikely to reflect a systematic software bias but is primarily shaped by phylogenetic divergence. As an evolutionarily ancient bryophyte species, *R. laxelimbatum* exhibits conserved organellar genomic features and a slow evolutionary rate at the DNA sequence level. To stabilize normal gene expression and physiological functions, this species may retain a high abundance of RNA editing events, resulting in more editing sites than those observed in angiosperms. However, the molecular mechanisms underlying the excessive accumulation of chloroplast RNA editing sites in *R. laxelimbatum*, and the intrinsic correlation between these unique editing patterns remain unclear. In both the mitochondrial and chloroplast genomes, the predominant type of amino acid conversion following RNA editing was the transition from hydrophilic to hydrophobic amino acids. This finding aligns with those of previous studies on other plant species (Chen et al. 2025). The alterations in protein functional properties caused by such transitions and their effects on ecological adaptation require further assessment.

### 4.3 Codon Usage Analysis of the *R. laxelimbatum* Organellar Genomes

The GC content at three codon positions in both the mitochondrial and chloroplast genomes exhibited a consistent trend, with the highest GC value at the first codon position, followed by the second and third positions. This finding aligns with previous studies on other terrestrial plants (Wang et al. 2026). Neutrality plot analysis revealed no significant correlation between GC12 and GC3 for mitochondrial protein‐coding genes, indicating a lack of coordinated base variation between the first, second, and third codon positions, reflecting the strong constraints imposed by natural selection on the mitochondrial codon composition. In contrast, a significant correlation between GC12 and GC3 was observed in the chloroplast genome, indicating that mutation pressure significantly contributes to shaping the chloroplast codon usage bias. However, nine A/U‐ending optimal codons were identified in the chloroplasts, demonstrating that translation‐associated natural selection is also involved, rather than being solely driven by mutation pressure.

The ENC‐plot further illustrated the divergent patterns of codon bias between the two organelles. Notably, the distribution range of ENC values was wider in the mitochondrial genome, with nearly all ENC values above 35. In contrast, chloroplast ENC values exhibited a narrower distribution range and smaller variation in codon bias but contained more genes with ENC values below 35 than the mitochondrion, indicating a stronger codon usage bias in a subset of chloroplast genes. PR2‐plot uncovered a distinct base‐composition imbalance at synonymous third codon positions: mitochondria exhibited T3s > A3s with comparable G3s and C3s, whereas chloroplasts exhibited T3s > A3s and G3s > C3s. Specifically, the most optimal codons in the mitochondria are C endings, inconsistent with the whole‐genome base composition background. This finding further supports the modulation of translational selection of highly expressed mitochondrial genes and highlights the distinct codon pattern of moss mitochondria compared with that of angiosperm organelles. The preference for A/T‐ending codons aligns with the higher AT content observed in chloroplast genomes, consistent with other plant lineages (Yan et al. 2026).

Correspondence analysis revealed that most genes were concentrated near the zero coordinate. However, several genes remained scattered across the plot, indicating that minor factors, in addition to the major drivers, contributed to codon variation. Correlation analysis of the primary codon usage parameters revealed that the second axis was significantly correlated with ENC and GC3s. This indicates that mitochondrial codon variation is primarily driven by the magnitude of codon bias and synonymous third‐position base composition. In chloroplasts, axis 1 was significantly correlated with CAI, FOP, GC3s, and GC content and exhibited a significant negative correlation with Gravy. These results indicate that chloroplast codon variation was affected by translational efficiency, base composition, and protein hydrophobicity. Collectively, the primary determinants of codon usage differ between the mitochondria and chloroplasts.

In summary, codon usage bias in both the mitochondrial and chloroplast genomes of this species is jointly shaped by mutation pressure and natural selection, whereas their relative contributions and major regulatory factors vary significantly. Mitochondria are subjected to stronger constraints from natural selection, with codon variations primarily governed by ENC and GC3s. In chloroplasts, mutation pressure plays a more significant role, whereas translational selection, base composition, and protein physicochemical properties also contribute to the formation of codon preferences. This divergence indicates that mitochondria and chloroplasts possess distinct replication‐repair machinery, tRNA pools, and translational systems and have experienced different evolutionary constraints. Studying codon bias and the influencing factors of species or groups can enhance understanding of genetic evolution in organisms and can serve as a reference for optimizing expression systems and producing metabolites with pharmacological implications (Wang et al. 2026; Zhang et al. 2026).

## 5 Conclusions

This study presents the entire mitochondrial genome of *R. laxelimbatum*, elucidating its genome size, GC content, gene composition, intron profile, repetitive sequences, and SSR loci. A partial comparative analysis from a dual‐organellar perspective revealed that the predicted RNA editing sites in the chloroplast genome of *R. laxelimbatum* far exceeded those in the mitochondrion, whereas the overall codon usage bias among the organelles was relatively weak and was regulated by multiple evolutionary factors. These findings provide crucial theoretical support for further elucidating the post‐transcriptional regulatory differences between the chloroplasts and mitochondria of this species and understanding the evolutionary mechanisms of organellar genomes.

## Author Contributions

**Jingyuan Niu:** conceptualization (lead), data curation (equal), formal analysis (lead), writing – original draft (lead), writing – review and editing (lead). **Shuo Shi:** conceptualization (lead), data curation (supporting), formal analysis (supporting), resources (lead), writing – original draft (supporting), writing – review and editing (supporting). **Shuangling Li:** data curation (equal), formal analysis (supporting). **Jiancheng Zhao:** conceptualization (supporting), resources (lead), writing – original draft (supporting). **Lin Li:** conceptualization (equal), data curation (supporting), resources (lead), writing – original draft (supporting), writing – review and editing (supporting).

## Funding

This work was supported by the Doctoral Scientific Research Activation Foundation of Henan Medical University (No. 505411).

## Conflicts of Interest

The authors declare no conflicts of interest.

## Data Availability

The organellar genome sequence data that support the findings of this study are openly available in GenBank of NCBI at (https://www.ncbi.nlm.nih.gov/) under the accession number MW147233 (chloroplast) and OL754429.1 (mitochondrion). The associated BioProject, SRA, and Bio‐Sample number are PRJNA725408, SRR14332107, and SAMN18878621.
